# Supplementary figures and images for: Widespread mitovirus sequences in plant genomes
Source: PeerJ. 2015 Apr 9;3:e876. doi: 10.7717/peerj.876 (PMC4393810; doi:10.7717/peerj.876)

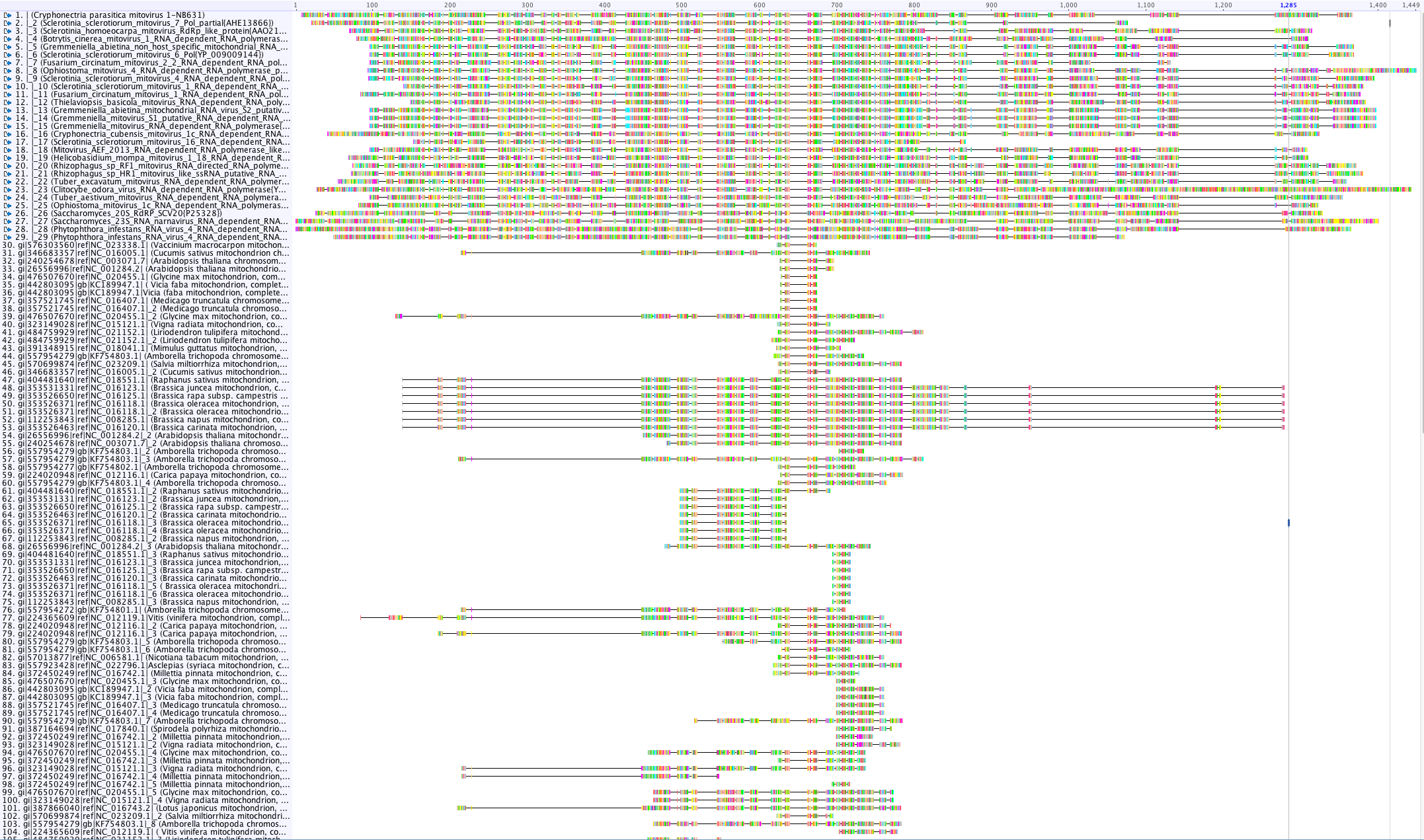

Supplement: Figure S1A — A FASTA version of this alignment is available on request. [file peerj-03-876-s001.png]

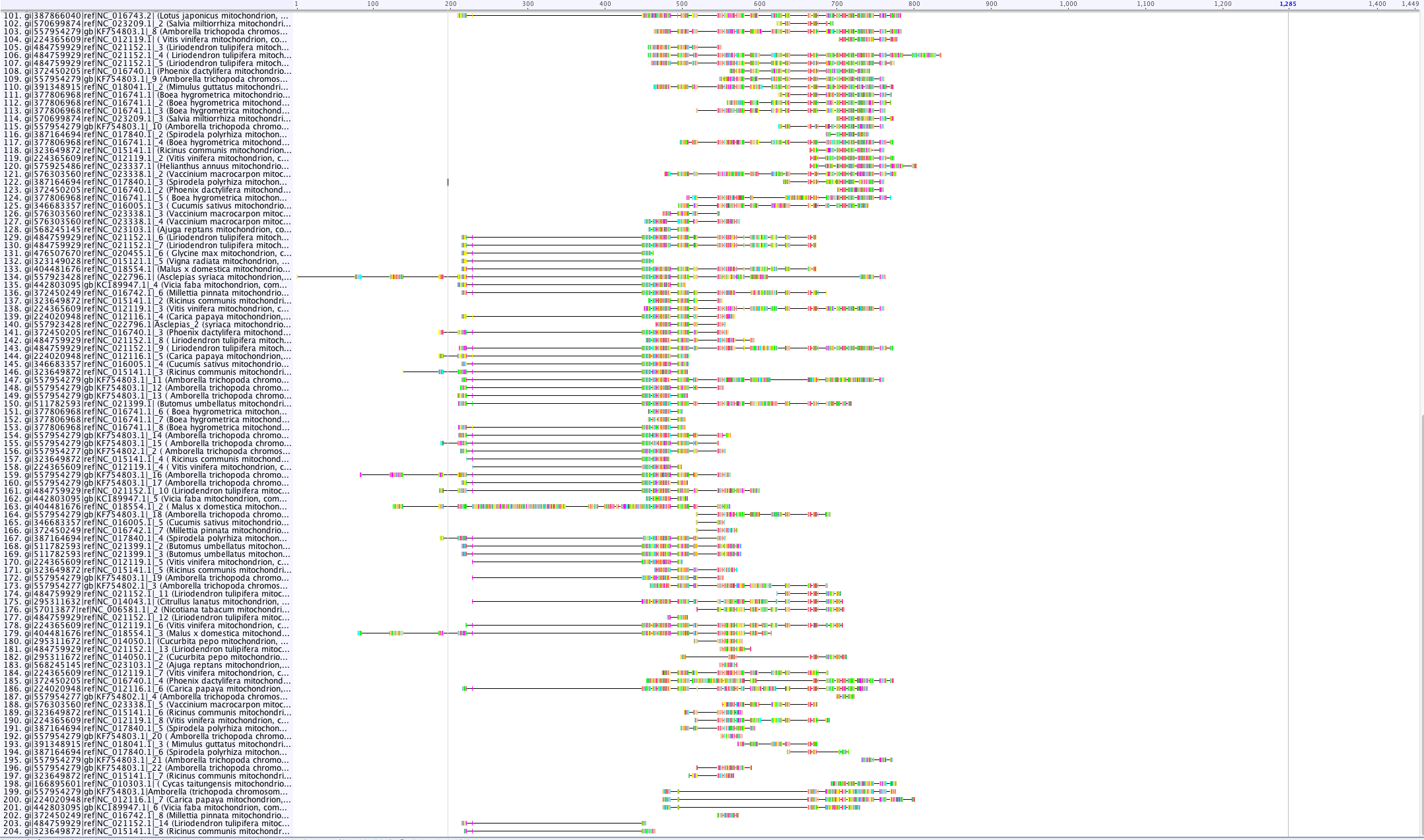

Supplement: Figure S1B — A FASTA version of this alignment is available on request. [file peerj-03-876-s002.png]

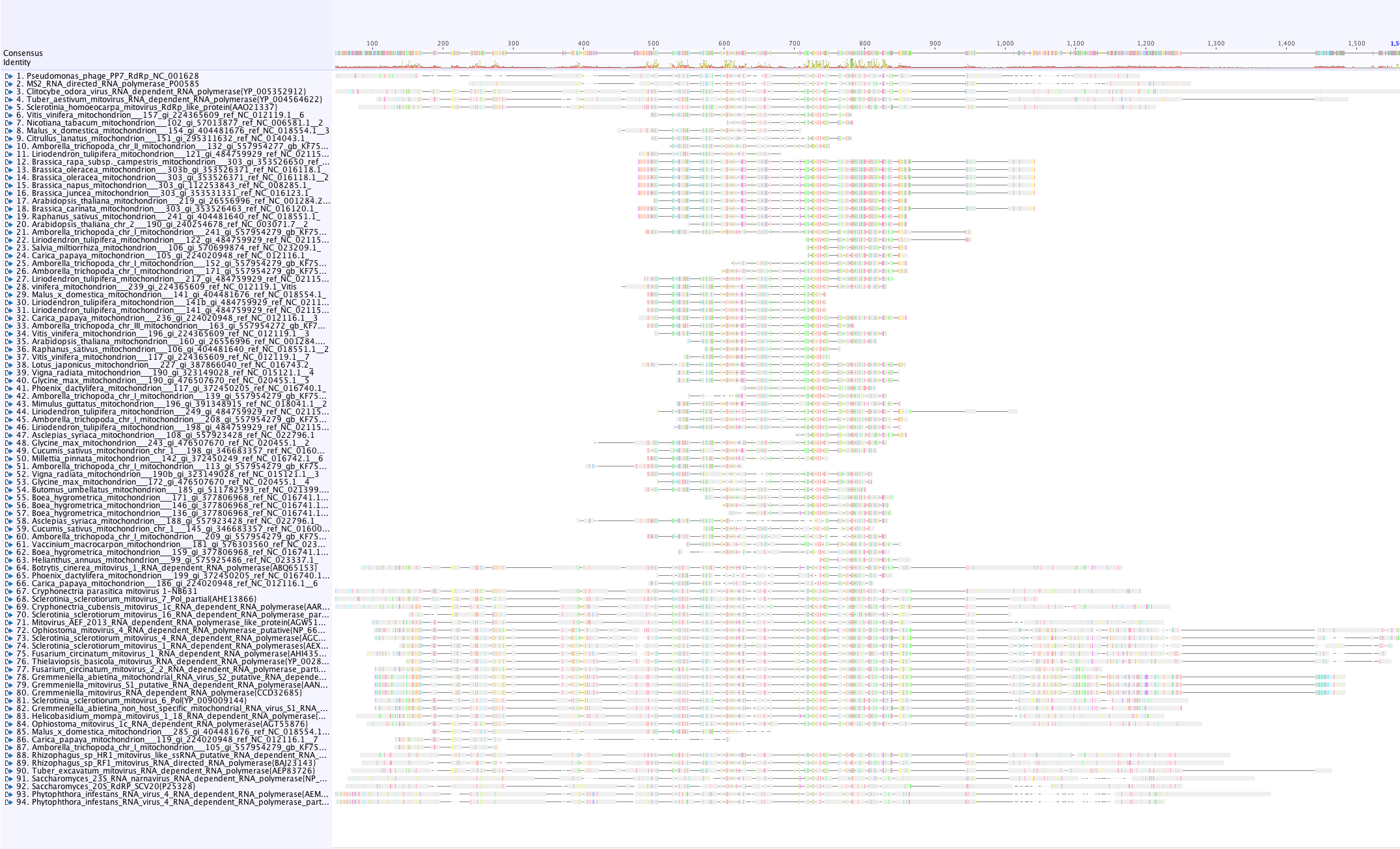

Supplement: Figure S2 — This is the alignment used to generate the cladogram of Fig. 4. A FASTA version of this alignment is available on request. [file peerj-03-876-s004.png]
