# Supplementary material for: Widespread mitovirus sequences in plant genomes
Source: PeerJ. 2015 Apr 9;3:e876. doi: 10.7717/peerj.876 (PMC4393810; doi:10.7717/peerj.876)
Supplement: Figure S3 [file peerj-03-876-s005.docx]

Sorghum bicolor chromosome 6, whole genome shotgun sequence

Sequence ID: [ref|NC_012875.1|](http://www.ncbi.nlm.nih.gov/nucleotide/242077817?report=genbank&log$=nuclalign&blast_rank=1&RID=M8FFJZJ9014)Length: 62208784Number of Matches: 2

Related Information

[Map Viewer](http://www.ncbi.nlm.nih.gov/mapview/maps.cgi?maps=blast_set&db=chromosome&na=1&gnl=ref%257CNC_012875.1%257C&gi=242077817&term=242077817%5Bgi%5D&taxid=4558&RID=M8FFJZJ9014&QUERY_NUMBER=1&log$=nuclalign)-aligned genomic context

Range 1: 19826538 to 19827041[GenBank](http://www.ncbi.nlm.nih.gov/nucleotide/242077817?report=genbank&log$=nuclalign&blast_rank=1&RID=M8FFJZJ9014&from=19826538&to=19827041)[Graphics](http://www.ncbi.nlm.nih.gov/nuccore/242077817?report=graph&rid=M8FFJZJ9014%5B242077817%5D&tracks=%5Bkey:sequence_track,name:Sequence,display_name:Sequence,id:STD1,category:Sequence,annots:Sequence,ShowLabel:true%5D%5Bkey:gene_model_track,CDSProductFeats:false%5D%5Bkey:alignment_track,name:other%20alignments,annots:NG%20Alignments%7CRefseq%20Alignments%7CGnomon%20Alignments%7CUnnamed,shown:false%5D&v=19826513:19827066&appname=ncbiblast&link_loc=fromHSP) Next Match Previous Match

| Alignment statistics for match #1 | | | | | | |
| --- | --- | --- | --- | --- | --- | --- |
| **Score** | **Expect** | **Method** | **Identities** | **Positives** | **Gaps** | **Frame** |
| 39.3 bits(90) | 0.26 | Compositional matrix adjust. | 37/173(21%) | 61/173(35%) | 35/173(20%) | -3 |

Features:

[533802 bp at 5' side: hypothetical protein](http://www.ncbi.nlm.nih.gov/nucleotide/242077817?report=gbwithparts&from=19292529&to=19292736&RID=M8FFJZJ9014)[859291 bp at 3' side: hypothetical protein](http://www.ncbi.nlm.nih.gov/nucleotide/242077817?report=gbwithparts&from=20686332&to=20686659&RID=M8FFJZJ9014)

Query 346 LLVDRDYYLPRWSGYAR--------------DSKVRYAVGQPMGALSSWAMLALTHHMIV 391 L+V R + P WS Y + R A+ P G + ++ H + Sbjct 19827041 LVVTRKSHSPTWSAYHKL*HLTQPDHLSLFTSHSTRVALRDPRGVSTIPLTISSPEHRTI 19826862

Query 392 QFAAAS----------------VGVTGWFKEYMVLGDDIVIYNSEVAKAYSTLMGTLGVG 435 A++ TG E++V D I N A +L T+G Sbjct 19826861 SLRASTESQATKPSRRWQPPRVTSTTGLQHEHLVPLDAISRSN-----ALESLTHTIG*S 19826697

Query 436 ISDTKSLTSKIGVFEFAKRLMDLEGPCQGLPLAEFAAARFNLSILFQSFRSRT 488

+S L + + + + + + PCQG P F A R L++ +S T

Sbjct 19826696 LSSICELETSLALPKHGH*VPRVLSPCQGRPQLLFIAPRAKLAVTLHCQKSTT 19826538
